# Can people self-select an exercise intensity sufficient to enhance muscular strength during weight training? A systematic review protocol of intervention studies

**DOI:** 10.1097/MD.0000000000017290

**Published:** 2019-09-20

**Authors:** Victor Hugo de Oliveira Segundo, Grasiela Piuvezam, Kesley Pablo Morais de Azevedo, Humberto Jefferson de Medeiros, José Carlos Leitão, Maria Irany Knackfuss

**Affiliations:** a Post-Graduate Program in Public Health; b Department of Public Health, Federal University of Rio Grande do Norte (UFRN), Natal/RN; c Department of Physical Education, University of the State of Rio Grande do Norte (UERN), Mossoró/RN, Brazil; d Center for Research in Sport, Health and Human Development, University of Trás-os-Montes and Alto Douro, Vila Real, Portugal.

**Keywords:** adherence, self-selected, strength training, systematic review, training load, weight training

## Abstract

**Background:**

Previous studies have reported that self-selection of the training intensity can be an interesting strategy to improve adherence in aerobic exercise programs. However, its effectiveness with weight training has not been systematically reviewed and remains unclear. In this study, we will describe a systematic review protocol that aims to investigate if people are able to self-select an intensity during weight training sufficient to enhance muscular strength.

**Methods:**

This protocol is guided by the Preferred Reporting Items for Systematic Reviews and Meta-Analyses Protocols. In this study, we will search the following electronic databases: PubMed, Science Direct, Scopus, Web of Science and SPORTDiscus. Intervention studies with at least one weight training session performed at self-selected intensity, with people from both genders and all age ranges will be included. The Downs & Black checklist will be used for methodological quality assessment. Two experienced reviewers will independently perform the selection of studies, data extraction, and evaluation of the methodological quality.

**Conclusion:**

This will be the first systematic review describing the results of weight training intervention studies with self-selected intensity. This study will provide high-quality and reliable evidence for health professionals and may direct methodological recommendations for further studies.

**PROSPERO registration number:**

CRD42019120323

## 1 Introduction

Regular physical activity represents a cornerstone in the primary prevention of at least 35 chronic diseases, even those that do not necessarily affect the locomotor system.^[1]^ In the United States, inadequate levels of physical activity have been associated with a significant percentage of health care expenditures.^[2]^ The same was observed in Brazil,^[3]^ Canada,^[4]^ the United Kingdom,^[5]^ China,^[6]^ and in at least 140 other countries.^[7]^

There is no doubt that exercise represents one of the most important strategies for the prevention of many diseases. This is the reason it has been called medicine, and has been increasingly recommended by health professionals.^[8]^ It is also known that low muscular strength is a strong predictor of mortality.^[9]^ Even though people know that exercise is good for health, however, a large proportion of the population remains physically inactive.^[10,11]^

Previous literature has shown that high rates of early dropout in exercise programs have an important impact on physical inactivity rates.^[11,12]^ In this sense, some studies have concluded that the loss of autonomy over the activity performed, such as the imposition of intensity by exercise professionals (especially the higher intensities), could have a significant impact on the feelings of pleasure/displeasure and result in early withdrawal from the exercise program.^[13–15]^

Following this conception, several studies have emerged testing the use of self-selected intensities and observed whether these intensities met those recommended by the main guidelines. A previous review proposed to analyze these studies and observe if these self-selected loads reached the intensities recommended by the guidelines.^[16]^ In most cases, self-selected intensities were in accordance with the guidelines. However, this review only included studies conducted with aerobic exercise.

In the last 5 years, several studies have been published testing this strategy in weight training.^[17–21]^ In studies with sedentary elderly, it was observed that they self-selected intensities according to the last guidelines.^[18,21]^ In a study by Elsangedy et al,^[19]^ sedentary male subjects selected intensities above the intensity suggested to increase their strength. However, in studies conducted with recreationally trained adults (minimum of 6 months of resistance training experience), it was observed that these loads were below the recommended intensity to enhance muscle strength.^[17,20]^

With this variety of results and the growing number of publications in recent years, it is important to systematically review the existing research on self-selected intensity during weight training. A systematic review is important for health professionals to help clarify what the literature is showing about this topic and to drive safer and more efficient decision-making. Therefore, the purpose of this paper is to describe a systematic review protocol that aims to investigate the existing research on self-selected intensity during weight training and identify if people select intensities that are conducive to enhance muscular strength according to current guidelines.

## 2 Methods

### 2.1 Protocol and registration

This protocol was prepared in accordance with the guidelines described by the Preferred Reporting Items for Systematic Reviews and Meta-Analyses Protocols (PRISMA-P).^[22]^

The protocol was registered with the International Prospective Register of Systematic Reviews (PROSPERO) on 05 April 2019 (CRD42019120323).

### 2.2 Inclusion criteria

For this review, articles that meet the eligibility criteria based on the study Population, Intervention, Comparison, Outcome and Study design (PICOS) will be included. The details are expressed in Table 1.

**Table 1:**
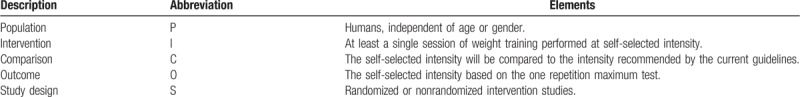
PICOS description.

Studies will be eligible for further analysis if the following inclusion criteria are met: original articles published in English language; intervention studies with at least 1 session of weight training performed at self-selected intensity; studies conducted with humans, regardless of gender and age group; and reported the self-selected intensity based on the one repetition maximum test.

### 2.3 Exclusion criteria

This will not be considered for analysis studies that used subjects with osteomyoarticular or intellectual problems, and studies that did not report clearly the physical activity level of participants.

### 2.4 Search methods for the identification of studies

A comprehensive search of the PubMed, Science Direct, Scopus, Web of Science, and SPORTDiscus databases will be conducted.

In each database, the title, abstract, and keywords search fields will be searched. The following terms will be used: “weight training,” “resistance training,” and “strength training,” in conjunction with such descriptors as “self-selected,” “self-regulated,” and “preferred.” The search equation was created based on the combination of OR and AND Boolean operators, according to the characteristics of each database. The search strategy details are presented in Table 2.

**Table 2:**
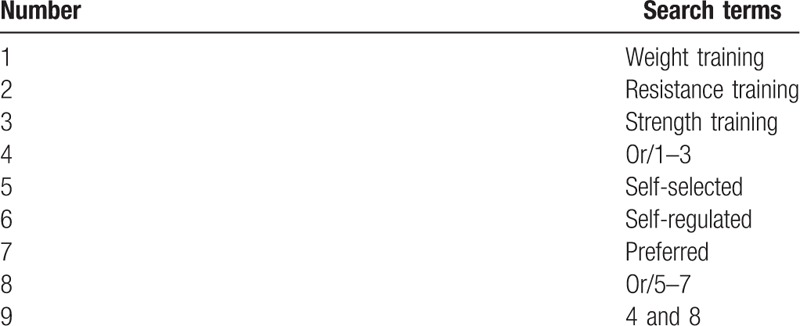
Search strategy applied in the databases.

Two reviewers will independently select all literatures according to the PRISMA flowchart and predesigned eligibility criteria. At the end of the database searches, the articles will be compiled into the EndNote bibliographical reference manager and duplicate articles will be removed.

**Figure 1:**
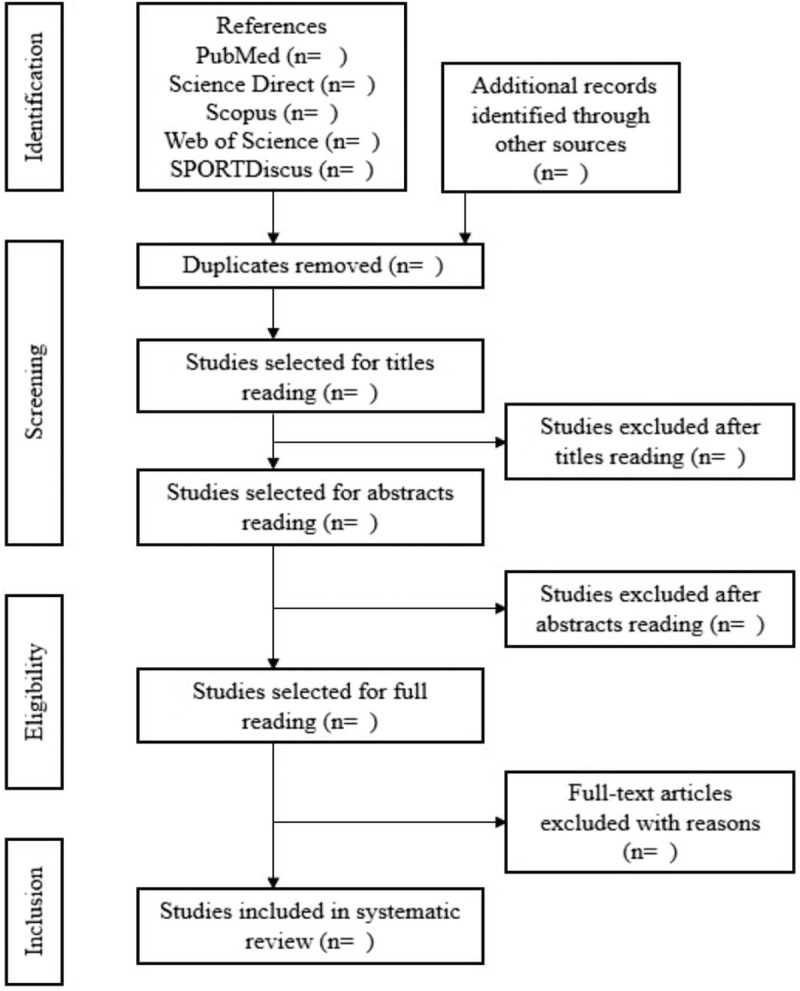
Article selection flowchart. Adapted from PRISMA-P.^[22]^

Titles and abstracts of identified articles will be checked for relevance in the first and second stages of screening, respectively. In the third stage, full-text articles will be retrieved and considered for inclusion. In addition, references cited in articles will be reviewed to locate any additional relevant articles not retrieved within the primary search (Fig. 1). Any divergences between 2 reviewers will be settled down by discussion with a third reviewer.

### 2.5 Quality assessment

To conduct an appraisal of the studies’ methodological quality, each of the included articles will be evaluated and allocated a score according the quality index for randomized and nonrandomized studies proposed by Downs and Black.^[23]^ Each published paper will be evaluated independently by 2 authors. To settle any disagreements in assigned scores, a third author will be consulted.

The quality index is a 26-item checklist including 5 subscales: reporting; external validity; internal validity—bias; internal validity—confounding; and power. Items are scored 0 or 1, except for 1 item in the reporting subscale, scored 0 to 2, and the single power item, scored 0 to 5. The total maximum score for quality is 32.

This quality index has demonstrated high internal consistency (Kuder–Richardson 20: 0.89), good test–retest (r = 0.88) and inter-rater (r = 0.75) reliability, and high correlations (r = 0.86–0.90) with other validated quality assessment instruments.^[23]^

### 2.6 Data extraction

Two reviewers will independently extract data by using a predefined standard data extraction form. This will be extracted information about the study (author and year of publication), participant characteristics (n°, sex, age and resistance training experience), methods (design of interventions and exercises), outcome measurements, and main findings.

Sub-analyses on age range and level of experience in weight training will be performed. All disagreements regarding the data extraction will be handled by discussion with a third reviewer.

## 3 Discussion

To our knowledge, this is the first systematic review that will summarize the findings on self-selected intensity during weight training. In a previous review investigating this strategy during aerobic exercise, the results showed that, in most cases, people self-selected intensities in accordance with the guidelines.^[16]^ It is important to mention that be free to choose the training intensity may play a role in the activation of brain reward systems and can, consequently, induce higher adherence rates.^[24]^ This is explained by the self-determination theory.^[25]^

Recent studies conducted with sedentary elderly showed that their self-selected intensity during weight training was just within current recommendations.^[18,21]^ However, another study with sedentary elderly women found intensities less than those recommended for improvements in muscle strength.^[26]^ The same was observed in most of the exercises in a study performed with sedentary adolescent girls.^[27]^

In studies with resistance-trained people (at least 12 months of experience with weight training), the self-selected loads were lower than those recommended for eliciting strength gains.^[17,20]^ These studies consisted of only 1 experimental session. In the study of Faries and Lutz^[28]^ that lasted 6 weeks, the authors observed that in the fifth training session, the loads reached those recommended by the guidelines. This finding reinforces the idea that even if self-selected loads are initially low, they are likely to quickly increase.

It has already been highlighted that individuals differ greatly in the levels of intensity they self-select. Consequently, some may choose intensities that are too low to be effective or too high to be safe.^[16]^ The study by Elsangedy et al^[26]^ showed these differences not only between individuals but also between types of exercise.

In studies with young adults, trained men^[29]^ and women^[30]^ self-selected loads below those recommended to enhance muscular strength. Conversely, sedentary men^[19]^ and women^[31]^ self-selected loads that met those recommended for novice individuals. These findings suggest that the level of experience in weight training can influence the individual's preferences. In addition, there is much heterogeneity in study designs with exercise training, as has been noted in other systematic review protocols.^[32,33]^

This wide range of information reinforces the need for a systematic review on the topic. It is also important not to lose sight of the impact these findings may have on public health spending. The protocol for this systematic review is presented in a clear and systematic way for the extraction of information and presentation of the findings. The results of this study will provide a summary of information and may benefit both health professionals and researchers.

## Author contributions

**Conceptualization:** Victor Hugo de Oliveira Segundo, Maria Irany Knackfuss.

**Data curation:** Victor Hugo de Oliveira Segundo, Grasiela Piuvezam, Kesley Pablo Morais de Azevedo.

**Formal analysis:** Victor Hugo de Oliveira Segundo, Grasiela Piuvezam, Kesley Pablo Morais de Azevedo.

**Investigation:** Victor Hugo de Oliveira Segundo, Kesley Pablo Morais de Azevedo, Humberto Jefferson de Medeiros.

**Methodology:** Victor Hugo de Oliveira Segundo, Grasiela Piuvezam, José Carlos Leitão, Maria Irany Knackfuss.

**Project administration:** Victor Hugo de Oliveira Segundo, Humberto Jefferson de Medeiros, Maria Irany Knackfuss.

**Supervision:** Grasiela Piuvezam, Humberto Jefferson de Medeiros, José Carlos Leitão, Maria Irany Knackfuss.

**Writing – original draft:** Victor Hugo de Oliveira Segundo, Kesley Pablo Morais de Azevedo.

**Writing – review & editing:** Grasiela Piuvezam, José Carlos Leitão, Maria Irany Knackfuss.

Victor Hugo de Oliveira Segundo orcid: 0000-0002-4596-9590.
